# Supplementary figures and images for: Paradox: Curcumin, a Natural Antioxidant, Suppresses Osteosarcoma Cells via Excessive Reactive Oxygen Species
Source: Int J Mol Sci. 2023 Jul 26;24(15):11975. doi: 10.3390/ijms241511975 (PMC10418684; doi:10.3390/ijms241511975)

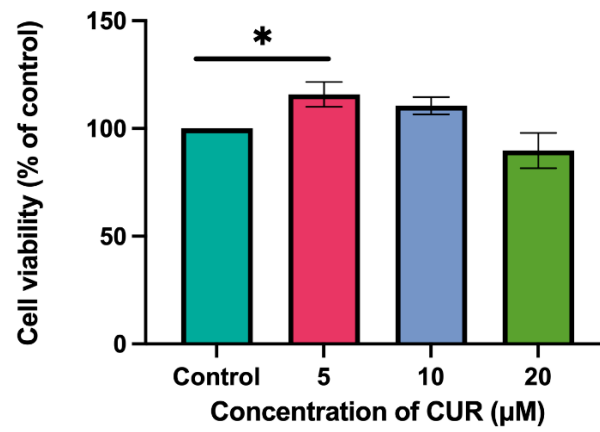

**Figure S1.** Viability of dental pulp stem cells treated by curcumin for 48 h.  $n = 3$  in each group, \*:  $p < 0.05$ .

Supplement: Supplementary file 1 [file ijms-24-11975-s001.zip › ijms-2502482-supplementary.pdf]
